# Effectiveness of Low-Dose Ketamine Infusion in Opioid Refractory Cancer Pain: A Case Report

**DOI:** 10.7759/cureus.31662

**Published:** 2022-11-18

**Authors:** Tuheen S Nath

**Affiliations:** 1 Surgical Oncology, Tata Medical Centre, Kolkata, IND

**Keywords:** opiods, iv ketamine, iv ketamine infusion, burst therapy, palliative care, ketamine burst therapy, refractory cancer pain, cancer pain, opioid-refractory pain, low-dose ketamine

## Abstract

Most patients with advanced cancer experience debilitating pain, which significantly affects their quality of life and has both physical and psychological implications. Opioids have been the mainstay of treatment for chronic cancer pain, but some people develop serious adverse effects or may become refractory to opioid use. There is always a need and search for alternative non-opioid analgesics with an acceptable safety profile, and one such drug is ketamine. In this era of evolving analgesic therapeutics, ketamine has been noted to have favourable results. Ketamine, a phencyclidine analogue, is an N-methyl-D-aspartate antagonist (NMDA), and it has been shown to have an analgesic effect at sub-anaesthetic doses by blocking NMDA-induced pain sensitization and enhancing opioid receptor sensitization. This is a case report of a 46-year-old Indian female with recurrent metastatic adenocarcinoma endometrium (International Federation of Obstetrics and Gynecology (FIGO) Grade II) involving the vaginal vault, rectum, and adrenal glands, along with para-rectal, bilateral iliac, and retroperitoneal nodal metastases, in which ketamine infusion was used successfully to alleviate the pain that was initially not controlled with an incremental dose of opioids. The patient presented with progressive pain in the peri-anal region, rated 8/10 on the Numerical Pain Rating Scale (NRS), following which she was treated with escalating doses of intravenous (IV) fentanyl, but with little to no relief. In view of the patient’s opioid-resistant pain, she was started on a low-dose ketamine IV infusion (50 mg in 50 ml of 0.9% NS) as "burst therapy," at infusion rates of 0.02 mg/kg/hr-0.08 mg/kg/hr, with adequate pain relief occurring at 0.08 mg/kg/hr. Literature suggests weight-based dosing of ketamine ranging from 0.06 mg/kg/hr to 0.8 mg/kg/hr was previously used to achieve satisfactory results. In this patient, even lower doses were effectively used to achieve optimum long-term analgesia, cause an upliftment in the patient’s overall mood and quality of life, and cause a significant reduction in opioid usage. However, further research is required to assess the efficacy of ketamine at such doses and its effect on opioid consumption. This case report will promote further study regarding optimum IV ketamine dosing and administration in the management of opioid-refractory pain in cancer patients, especially in the Indian population.

## Introduction

A large proportion of patients with cancer experience pain secondary to their disease, which has a significant impact on their quality of life. In a meta-analysis of 122 studies on cancer pain, Marieke et al reported that the pain prevalence rate was 66.4% in metastatic or terminal disease, while moderate to severe pain was reported by 38% of all patients [1]. While opioids are considered the main pillars of treatment in the palliative setting, about 20% of patients have persistent or refractory pain despite aggressive up-regulation of doses or may develop opioid tolerance after chronic treatment [2]. Also, long-term use of opioids is linked to numerous somatic as well as neuropsychiatric side effects. All these factors warrant the need for an alternative drug or mechanism of analgesia, and one such alternative is low-dose ketamine. This report presents a case of metastatic endometroid adenocarcinoma (FIGO Grade II) where a low-dose ketamine infusion as "burst therapy" was used to successfully alleviate the pain that was not controlled with an incremental opioid-based pain management regime.

## Case presentation

Here we present a case of a 46-year-old female with recurrent metastatic adenocarcinoma of the endometrium (FIGO Grade II) involving the vaginal vault, rectum, and adrenal glands along with para-rectal, bilateral iliac, and retroperitoneal nodal metastasis, who was admitted to the palliative department with a chief complaint of fever, abdominal distention, and uncontrolled pain. The patient underwent a total laparoscopic hysterectomy with bilateral salpingo-oophorectomy and pelvic lymphadenectomy about three years ago and a palliative diversion loop sigmoid colostomy owing to her advanced rectal disease with obstructive symptoms six months ago. She had also received three cycles of adjuvant chemotherapy with carboplatin and paclitaxel one year ago, owing to recurrence evidenced on PET-CT. She had progressive pain around her perianal region radiating to the lower back for the last six months, which was being controlled with a varying combination of analgesics, including pregabalin, tapentadol, etoricoxib, paracetamol, and morphine. She had no other comorbidities and no prior history of drug or alcohol dependence.

On presentation, her pain score was 8/10 (according to the Numerical Pain Rating Scale), while she was on an out-patient regimen of oral pregabalin 150 mg by mouth twice daily, oral paracetamol 1000 mg by mouth thrice daily, and oral morphine 10 mg by mouth every four hours with a 10 mg morphine pro re nata (PRN) dose for spikes of pain (approximately 60 mg total oral morphine equivalent (OME) ). Upon examination, the patient was alert, conscious, and cooperative, with an Eastern Cooperative Oncology Group (ECOG) performance score of 2/3. She was febrile and appeared toxic. On general examination, there was bilateral pedal pitting oedema, no pallor, no cyanosis, no icterus, and no palpable lymphadenopathy. Her PER-abdominal examination showed a distended, non-tender abdomen that was tympanic on percussion. A complete blood count showed a total leukocyte count (TLC) of 18500 with an absolute neutrophil count (ANC) of 15500. Given her clinical presentation, she was started on IV antibiotics, IV fluids, and other supportive medications. Subsequent blood and urine culture reports were negative for any growth. For her pain relief, she was initially started on a continuous IV infusion of fentanyl at 30 mcg/hr along with adjuvant analgesics, including oral pregabalin 300 mg per day and oral paracetamol three grammes per day. A medical oncology opinion was also sought, but there was no role for palliative chemotherapy due to the advancement of the disease. The patient was admitted with the best supportive care.

Within the first 12 hours, the patient received 360 mcg of IV fentanyl (36 mg OME) with inadequate pain relief, still reporting a pain score of 8-9 on a scale of 10. She described the pain as an "intolerable throbbing" or "burning sensation" in the perianal region, sometimes radiating to the lower back. The fentanyl infusion rate was thus increased to 40 mcg/hr, along with additional PRN dosing to be given as and when necessary. During the next 24 hours, there was a 100% increment in Fentanyl dosing (145 mg OME) with a reduction of 1/10 in the pain score. On day three of hospitalization, she continued to have escalating opioid needs, as evidenced by an increase in IV Fentanyl infusion to 75 mcg/hr and increasing demand for PRN doses every four hours and sometimes even hourly, totalling 2250 mcg in 24 hours (225 mg OME). However, the pain control was still inadequate, with an average persistent pain score of 7.5/10 and slight somnolence, and she also started exhibiting adverse effects of opioids, including nausea, constipation, lethargy, and ascending drug tolerance. A radiotherapy opinion was also sought regarding pain management and slight stomal bleeding, but it was agreed that there was no role for palliative radiotherapy due to extensive disease and nerve involvement and that it was in the patient's best interest to continue supportive care under the palliative team. The multidisciplinary palliative care team decided on such clinical grounds not to further escalate the opioid treatment, and the patient was considered for a low-dose ketamine infusion.

As per local and institutional guidelines, a low-dose ketamine infusion known as "burst therapy" was started as an adjunct to the opioid treatment at a standard concentration of 50 mg in 50 ml of 0.9% normal saline (1 mg/ml) at a rate of 1 ml/hr, i.e., 0.02 mg/kg/hr. Observational data were collected during the entire ketamine infusion duration, including pain characteristics, a pain score (NRS) every four hours, adverse effects, and total opioid usage [Table 1]. During the first 12 hours of the low-dose ketamine infusion, the patient reported a pain score of 6/10, and the requirement for the bolus fentanyl administration had reduced. In the following 24 hours (day 2 of the ketamine infusion), the rate of infusion was increased to 3 ml/hr, i.e., 0.06 mg/kg/hr, and the patient reported an average pain score of 4.5/10 with only one PRN dose of IV fentanyl required. Subsequently, on the next day (day three of the ketamine infusion), the rate was up-titrated to 4 mL/hr, i.e., 0.08 mg/kg/hr, which showed the most effective pain reduction at 1-2/10 and no requirement for SOS fentanyl. The team then decided to discontinue the IV fentanyl infusion and switch the patient to a 50 mcg/hr fentanyl transdermal patch. Day four yielded similar results, with an average pain score of 1/10 and patient satisfaction. These results manifested alongside a 46.66% reduction in opioid usage (-105 OME) and the mitigation of opioid-related side effects, including opioid dependence and tolerance. The patient had also been closely monitored for ketamine-induced side effects, including hallucinations, palpitations, ataxia, and disorientation, but she did not report any. Following that, the patient reported feeling less pain than before, the ketamine infusion was discontinued, and the patient was discharged on a fentanyl patch of 50 mcg, oral pregabalin 75 mg twice daily, and oral diclofenac 75 mg twice daily. On review after 14 days, the patient reported a relatively pain-free status (0/10) and also had significant improvement in her quality of life and activities of daily living.

**Table 1:** Average pain score and OME before and after low-dose IV ketamine infusion. N/A: not applicable; IV: intravenous This table was adapted from reference [3] and was created by the first author.

Day	IV ketamine infusion rate	Oral morphine equivalent (OME)	% Change in OME from day 0	Average pain Score	% Reduction in pain score from day 0
Day 0 (Prior to ketamine infusion)	N/A	225 mg	N/A	7.5	N/A
Day one	0.02 mg/kg/hr	215 mg	-4.44%	6	20%
Day two	0.06 mg/kg/hr	190 mg	-15.55%	4.5	40%
Day three	0.08 mg/kg/hr	180 mg	-20%	1.5	80%
Day four	0.08 mg/kg/hr	120 mg	-46.66 %	1	86.66%

**Figure 1:**
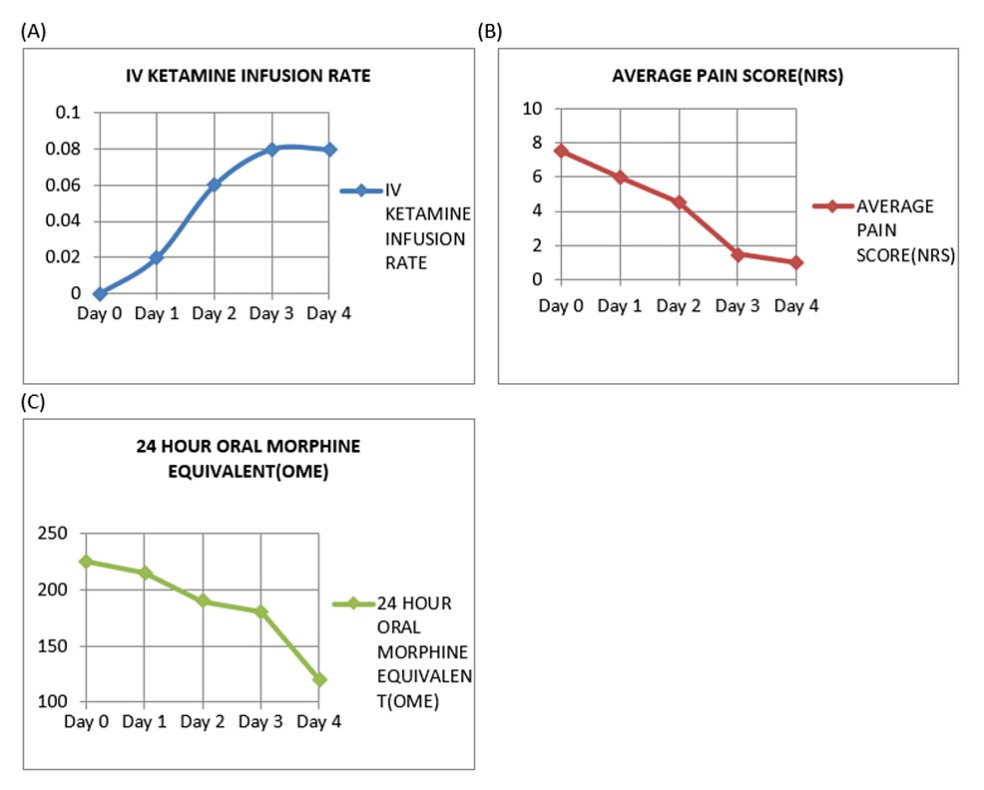
Pain medication and pain score analysis during low-dose IV ketamine infusion (A) IV ketamine dosing (mg/kg/hr); (B) average pain score (n/10); (C) 24-hour oral morphine equivalent (mg). NRS: numerical rating scale, OME: oral morphine equivalent; IV: intravenous This figure was adapted from reference [3] and was created by the first author.

Overall, the results of the low-dose ketamine intravenous infusion as burst therapy in this patient demonstrate its usefulness in the palliative care setting.[Figure 1] It not only provided adequate pain relief but also reduced total opioid usage, alleviating its associated side effects such as dependence and tolerance, and had a significant positive impact on the patient's mood and overall well-being.

## Discussion

Pain due to cancer, especially in advanced or metastatic disease, may have various aetiologies and underlying pathological mechanisms, and correctly understanding them will provide the most efficient and tailor-made treatment strategy. Mainly, they can be classified as nociceptive, neuropathic, and mixed types of pain with associated hyperalgesia and allodynia [4]. In this case, the patient was likely suffering from mixed nociceptive and neuropathic pain initially. It is widely established that opioids are the first-line drugs for the treatment of this type of pain, but in this case, long-term opioid therapy and rapid up-titration of doses might have led to opioid-induced hyperalgesia (OIH) [5]. OIH is defined as a state of opioid-induced nociceptive sensitization (peripheral and central) [6]. Although the exact mechanism of action of OIH is not well known, various studies have reported that NMDA (N-methyl D-aspartate) receptor activation plays a role in the mechanism of hyperalgesia and allodynia and reduces opioid sensitization [6-9]. The treatment plan in such cases should include the prevention of further escalation of opioid dosage, a de-escalation if possible, and the administration of alternative agents, preferably NMDA receptor modulators. Ketamine, which gained popularity since the 1980s as a general anaesthetic, is an NMDA antagonist, and at sub-anaesthetic doses it has shown to have an analgesic effect by blocking NMDA-induced pain sensitization and enhancing opioid receptor sensitization, thereby justifying its effectiveness in this case.

Regarding the optimal dosing, previous literature on IV ketamine has suggested weight-based dosing starting at 0.06 mg/kg/hr and increasing to 0.8 mg/kg/hr to achieve satisfactory results [3,10]. Brockett et al. conducted a recent randomized, double-blind, placebo-controlled trial to assess the efficacy of low-dose ketamine as an adjunct to opioids in pain management. Participants were divided into two groups, one receiving IV ketamine at a rate of 0.1 mg/kg and the other receiving normal saline in addition to the IV opioid medication. The results showed a significant reduction in pain scores in the ketamine group [11].

In this patient, however, considering the relatively lower initial pain score and already existing drowsiness, the palliative care team decided to start the IV ketamine infusion as "burst therapy" at 0.02 mg/kg/hr, with ideal pain relief occurring at 0.08 mg/kg/hr. "Burst therapy" refers to the usage of ketamine as a continuous IV infusion for a few days followed by a sudden cessation, carrying the same similitude as a burst therapy of steroids. Although there are not many studies on ketamine as "burst therapy," the findings of this study corroborate with a similar study conducted by Jackson et al on 39 cancer patients where ketamine (100 mg-500 mg/day) was successfully used as a brief burst to confer long-term analgesia in refractory pain [12].

The results of this study showed that the infusion of IV ketamine at these lower infusion rates resulted in significant pain reduction (-86.66%), improvement of the patient’s overall mood, and a noteworthy reduction in opioid consumption (-46.66%). In addition, she seemed much more jovial and optimistic than before, as noted by the doctors and nurses during rounds, thus supporting previous evidence on the role of ketamine in treating depression in patients with advanced cancer [13,14]. Furthermore, on review after two weeks, the patient reported a pain-free status, demonstrating the effectiveness of the low-dose "burst therapy" of ketamine beyond the infusion period also.

All aspects of the study were in accordance with ethical standards, and informed consent was obtained from the patient for the same.

## Conclusions

Chronic pain management in terminal cancer patients constitutes one of the most important yet difficult aspects of the cancer care continuum. A large proportion of patients with advanced disease experience excruciating pain, and while physicians consider opioid analgesics as the first line of treatment, they are not always effective and may lead to various side effects, including tolerance. Here we document the case of a patient in whom a low-dose ketamine infusion was used as "burst therapy" to achieve optimum long-term analgesia and an associated improvement of mood, which was not possible after initial opioid-based management. However, further research is required to assess the efficacy of ketamine to reduce opioid consumption and opioid tolerance or dependence.

## References

[1] van den Beuken-van Everdingen MH, Hochstenbach LM, Joosten EA, Tjan-Heijnen VC, Janssen DJ (2016). Update on prevalence of pain in patients with cancer: systematic review and meta-analysis. J Pain Symptom Manage.

[2] Waldfogel JM, Nesbit S, Cohen SP, Dy SM (2016). Successful treatment of opioid-refractory cancer pain with short-course, low-dose ketamine. J Pain Palliat Care Pharmacother.

[3] Amin P, Roeland E, Atayee R (2014). Case report: efficacy and tolerability of ketamine in opioid-refractory cancer pain. J Pain Palliat Care Pharmacother.

[4] Caraceni A, Shkodra M (2019). Cancer pain assessment and classification. Cancers (Basel).

[5] Mercadante S, Arcuri E, Santoni A (2019). Opioid-induced tolerance and hyperalgesia. CNS Drugs.

[6] Lee M, Silverman SM, Hansen H, Patel VB, Manchikanti L (2011). A comprehensive review of opioid-induced hyperalgesia. Pain Physician.

[7] Dickenson AH (1994). Neurophysiology of opioid poorly responsive pain. Cancer Surv.

[8] Mayer DJ, Mao J, Price DD (1995). The association of neuropathic pain, morphine tolerance and dependence, and the translocation of protein kinase C. NIDA Res Monogr.

[9] Bell RF, Eccleston C, Kalso EA (2017). Ketamine as an adjuvant to opioids for cancer pain. Cochrane Database Syst Rev.

[10] Pharo GH, Zhou L (2007). Controlling cancer pain with pharmacotherapy. J Am Osteopath Assoc.

[11] Brockett-Walker C (2019). The use of ketamine as an adjunct to treating opioid refractory cancer-related pain in the emergency department. Adv Emerg Nurs J.

[12] Jackson K, Ashby M, Martin P, Pisasale M, Brumley D, Hayes B (2001). "Burst" ketamine for refractory cancer pain: an open-label audit of 39 patients. J Pain Symptom Manage.

[13] Sexton J, Atayee RS, Bruner HC (2018). Case report: ketamine for pain and depression in advanced cancer. J Palliat Med.

[14] Stefanczyk-Sapieha L, Oneschuk D, Demas M (2008). Intravenous ketamine "burst" for refractory depression in a patient with advanced cancer. J Palliat Med.

